# Personalising Management of Behavioural and Psychological Symptoms of Dementia in Nursing Homes: Exploring the Synergy of Quantitative and Qualitative Data

**DOI:** 10.1155/2020/3920284

**Published:** 2020-07-09

**Authors:** Gubing Wang, Haotian Gong, Armagan Albayrak, Tischa J. M. van der Cammen, Gerd Kortuem

**Affiliations:** ^1^Faculty of Industrial Design Engineering, Delft University of Technology, Delft 2628CE, Netherlands; ^2^Section of Geriatric Medicine, Department of Internal Medicine, Erasmus Medical Center, University Medical Center Rotterdam, Rotterdam 3015GD, Netherlands; ^3^Academic Department of Geriatric Medicine, Brighton and Sussex Medical School, Brighton BN19PX, UK

## Abstract

Researchers have been exploring how to manage Behavioural and Psychological Symptoms of Dementia (BPSD) in a personalised way, meanwhile, assistive technologies have been developed to collect a variety of personal data. This urges more research in investigating the combination of: data collected by the care team, which are mainly qualitative; and data collected by assistive technologies, the majority of which are quantitative. Previous studies, however, have yet to explore if and how a combination of quantitative and qualitative data could facilitate the care team to better understand each resident with dementia in the nursing home context for personalised BPSD management. Guided by a Research through Design approach, a prototype for collecting and visualising the quantitative and qualitative data towards personalised BPSD management was developed together with the care team. Via developing this prototype, knowledge was gained in what types of data could be combined for personalised BPSD management in nursing homes, what are their values, how to collect and present them, and how to introduce them in the working routine of the care team for analysis. The main findings suggest that the types of data to be collected could be unique for each resident with dementia; the quantitative and qualitative data are of value to each other during data collection and analysis; data collection should be quick and standardised yet flexible for the care team; the overview page is vital for data presentation; and user scenarios could be created to nudge the care team to analyse the data at certain points of their working routine. In general, a combination of qualitative data and quantitative data could help the care team to discover more insights about each resident with dementia and thus improve the current practice of personalised BPSD management.

## 1. Introduction

**Figure 1:**
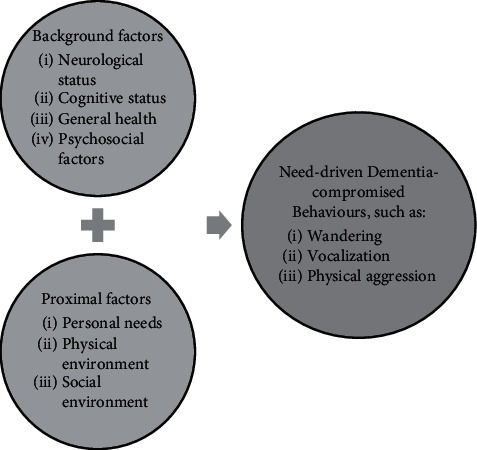
Need-driven Dementia-compromised Behaviour model (modified based on [4]).

Ninety-seven percent of People with Dementia (PwD) will develop at least one behavioural or psychological symptom over a five-year period [1], which includes agitation, aggression, depression etc. [2]. The collective term for these symptoms is Behavioural and Psychological Symptoms of Dementia (BPSD), which has been identified as a predictor of nursing home placement [3]. BPSD is regarded as a type of communication for PwD to express their unmet needs and goals [4–6]. According to the Need-driven Dementia-compromised Behaviour model (see Figure 1), the factors contributing to BPSD are divided into the background and proximal factors. Background factors consist of neurological status, cognitive status, general health, and psychosocial factors. These factors are inherent to the PwD thus difficult to change. Proximal factors include personal needs, physical environment and social environments. These factors could be modified for preventing or intervening BPSD. Each individual has his or her unique background and proximal factors; thus, the management of BPSD should be tailored to individual needs. However, the current personalised methods for managing BPSD are limited [7–9], which implies this field needs to be explored, especially in the nursing home setting.

The recent research for personalised BPSD management in nursing homes is on facilitating the creation of a personal care plan for each resident. For instance, in the Behaviour Analytics & Support Enhancement programme, an individual care plan was created for each PwD participant based on a multidisciplinary meeting [10]. This care plan making process only relies on qualitative data generated by the care team. A care team is composed of Professional Caregivers, e.g., nurses and care assistants, and Health Care Professionals, e.g., doctors, psychologists, dietitians, and managers. Professional Caregivers are distinguished from Health Care Professionals in this study for the different roles they play in the care plan making process. This is because Professional Caregivers are involved in caring activities on a daily basis; accordingly, they are more involved with the data collection for care plan making. The involvement of Health Care Professionals is less than that of Professional Caregivers; they normally meet up with Professional Caregivers to get second-hand information about the current mental states and needs of PwD before collecting data themselves. In general, mainly the Professional Caregivers observe and remember the behaviours of each PwD and discuss them in the multidisciplinary meeting with Health Care Professionals, so as to create the individual care plan. However, the qualitative data generated by the care team might not be accurate, since human memory can be influenced by a variety of factors, can fade quickly, and can be inadequate in remembering details [11]. It is challenging to collect and analyse data about care recipients who cannot confirm if the data collected and the analyses are correct.

Concurrently, a range of assistive technologies has been developed to assist BPSD management in nursing homes [12]. The commonalities shared by them are that objective data about PwD are automatically tracked by sensors and stored and processed by algorithms quantitatively, and the analysed outcomes are then presented to Health Care Professionals and Professional Caregivers via a network communication platform [13]. The types of data collected range from behavioural data, such as motion [14], acoustics [15], and location [16], which are used to monitor the behaviours of PwD; to physiological data, such as heart rate [17], skin conductance [18], and respiratory rate [19], which are used to evaluate the emotional state of PwD; to contextual data, such as location [20], temperature [21], and light level [21], which are used to assess the environments that the PwD is in.

Researchers in Data-enabled Design stressed the importance of combining quantitative data (referred to as “sensor data” in Data-enabled Design) and qualitative data, for designers to gain contextual, behavioural, and experiential insights in developing intelligent ecosystems [22]. A data framework on combining quantitative data and qualitative data for BPSD management has also been developed for researchers [21]. The researchers can use this framework to plan and organise data collection, extraction, and analysis to understand the context for dementia caregiver empowerment. More research is needed to explore if and how a combination of quantitative and qualitative data could facilitate the care team, instead of designers or researchers, to better understand PwD in the nursing home context for managing BPSD in a personalised way.

In this study, the combination of qualitative data collected by the care team and quantitative data collected by the Indoor Positioning System (IPS) was explored. This is because, among the objective quantitative data collected by assistive technologies, location data has been used for monitoring BPSD and is also recognised as central to the context in BPSD management [12]. Not only movement patterns but also other relevant parameters (e.g., travelled distance, interaction time with others) could be derived from the location data. Therefore, combining location data and qualitative data for BPSD management can be the starting point of exploration. The IPS has been developed to monitor the locations of objects and people over space in real-time in the indoor environment [23], which is suitable for this study given the fact that PwD involved in this study usually stay indoors due to their BPSD.

The hypothesis is that a combination of qualitative data generated by the team and quantitative data generated by the IPS could help the care team to discover more insights about a resident and thus improve the current practice of personalised BPSD management. This study is aimed at exploring what types of data can be combined for personalised BPSD management in nursing homes, what are their values, how to collect and present them, and how to introduce them in the working routine of the care team for analysis.

## 2. Materials and Methods

### 2.1. Study Design

The Research through Design approach was adopted whereby “design activities play a formative role in the generation of knowledge” [24]. This approach has been used in codesigning ambient assisted living environments for informal dementia care and yielded concrete insights on the attitude of informal caregivers towards these assistive technologies [25]. Through a series of design activities, e.g., interviews and evaluation sessions, a prototype was developed for combining the quantitative and qualitative data on personalised BPSD management with the care team. The abstract theory is that a combination of quantitative and qualitative data could facilitate personalised BPSD management, and this prototype could then enable the care team to gain experience in collecting and analysing these data. Therefore, the function of this prototype is to “connect abstract theories to experience” [24], which helps to generate knowledge regarding the four research questions in this study:

1. What types of data can be combined for personalised BPSD management?
2. What are the values of these combinations for personalised BPSD management?
3. How to collect and present these data?
4. How to introduce these data in the working routine of the care team for analysis?

### 2.2. Ethics Approval

The study was carried out in Zorggroep Elde nursing home in the Netherlands. In this nursing home, there is a special ward for PwD exhibiting BPSD. The study protocol was approved by the Human Research Ethics Committee of Delft University of Technology (see supplementary file for the Ethics Approval Letter of Delft University of Technology, 3 December 2018). Since this study involves collecting location data from the PwD and Professional Caregivers, written informed consent was obtained from Professional Caregivers and the legal representatives of PwD. In the end, the legal representatives of eight residents and all twelve Professional Caregivers have given consent to allow the IPS to collect their location data. The IPS requires each participant to carry a physical tag (in the shape of a thick ID card), and the location of the tag is tracked by the system for data collection. Three residents dropped out of the study because they disliked the tags, and their data were excluded from the study accordingly. The participants involved in the location data collection are shown in Table 1.

### 2.3. Research through Design Process

**Figure 2:**
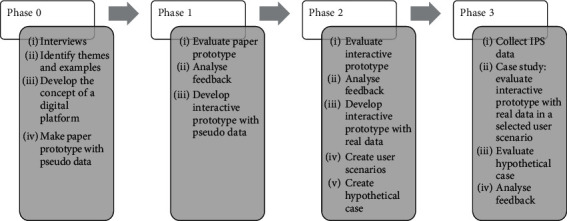
Activities of each phase in the Research through Design process.

The Research through Design process consists of four phases, and the learnings generated in the first phase act as the starting point of the next phase. Throughout the process, a prototype was developed and refined based on the feedback from each phase. The participants in each phase of the Research through Design process are shown in Table 2. Written informed consent was obtained from all participants. Some Professional Caregivers participated in both the location data collection and the Research through Design process. The aims of the four phases are to answer the four research questions, and the activities carried out in each phase are 2 summarised in Figure 2.

#### 2.3.1. Phase 0

**Figure 3:**
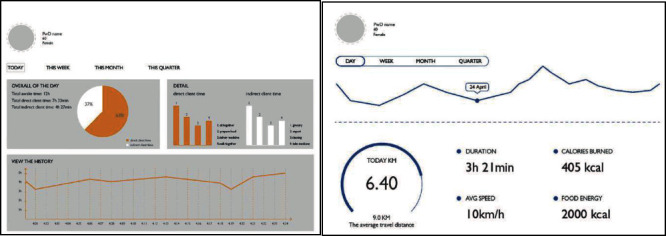
Interface pages of the paper prototype.

Semi-structured interviews were conducted with Professional Caregivers and the responsible doctor, the responsible manager, the responsible psychologist, and the responsible dietitian of the ward. The interviews are aimed at discovering concrete themes and examples of the proximal factors since the Need-driven Dementia-compromised Behaviour model has only provided abstract concepts about what proximal factors might be. The interview guide can be found in the supplementary files. In the interview guide, the Need-driven Dementia-compromised Behaviour model (see Figure 1) was used as a conversation starter. Each interview lasted 45-60 min and was audiotaped and transcribed verbatim. Thematic analysis was undertaken and followed the six steps recommended by Braun and Clarke [26]. Specifically, the first and second authors (GW and HG) familiarised with the data, generated initial codes, searched for themes, reviewed the themes, then defined and named the themes, and finally produced a report on themes and examples identified for the proximal factors. The types of data that could be combined were initialised based on the report. The concept of a digital platform was developed, and the interfaces of the digital platform were represented in a paper prototype to illustrate the concept. Some interface pages of the paper prototype are shown in Figure 3. Due to the time required for installing and adjusting the IPS, the data used for this prototype was pseudo data. The pseudo data were created based on the educated guess of the researchers via reading the reports in the ward.

#### 2.3.2. Phase 1

**Figure 4:**
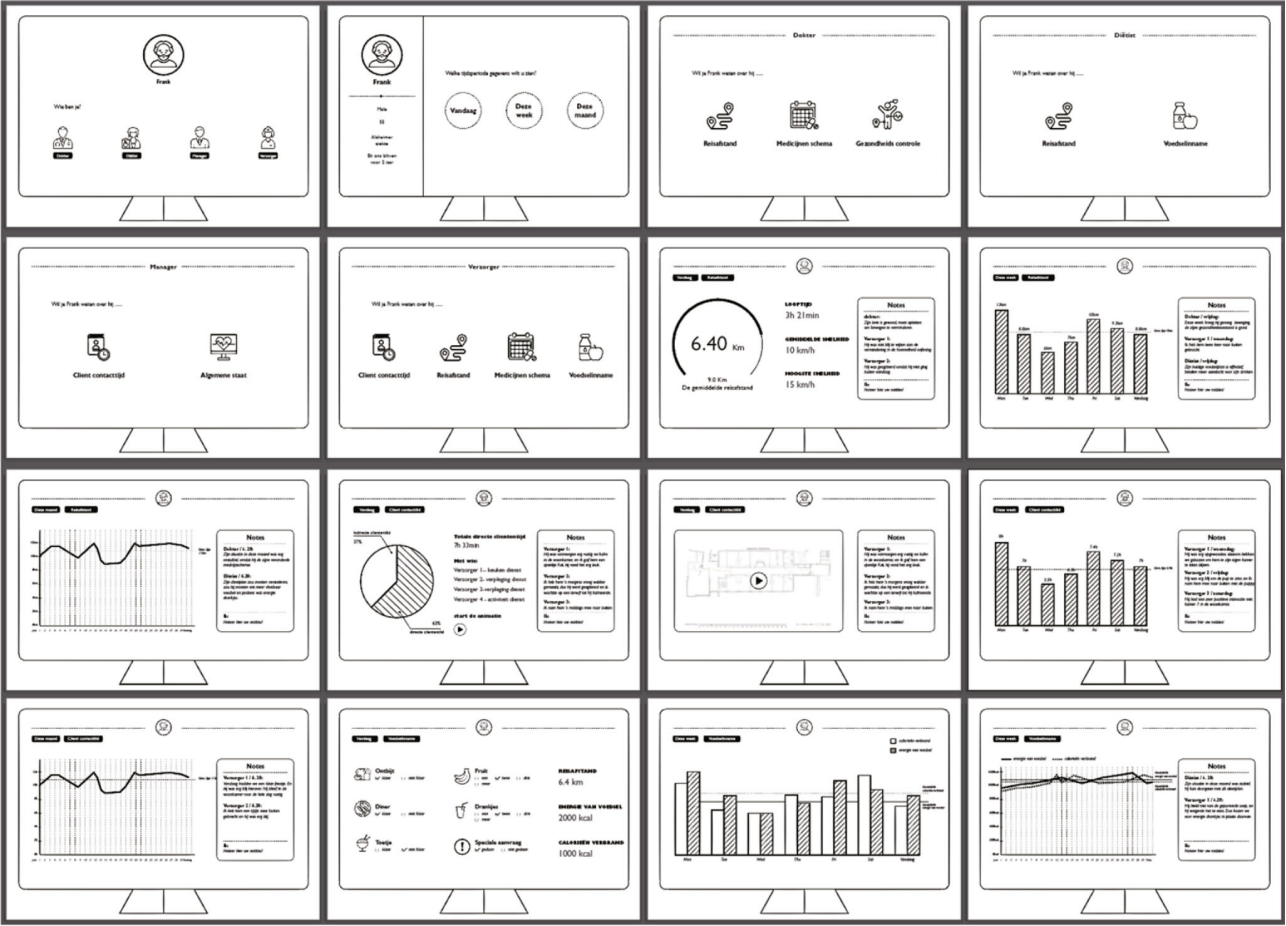
Interface pages of the interactive prototype.

The paper prototype was presented to participants for feedback on data visualisation and interface design via individual evaluation sessions with each session lasting for 15 min. The sessions were recorded and transcribed verbatim. The transcripts were analysed using affinity diagramming [27]. Based on feedback from the evaluation session, the concept was further developed and an interactive prototype was made accordingly. Some interface pages of the interactive prototype are shown in Figure 4. The data used for this prototype was the same pseudo data, since the researchers were still exploring data visualisation and interface design; whether the data is real would not affect the user feedback gathered.

#### 2.3.3. Phase 2

**Figure 5:**
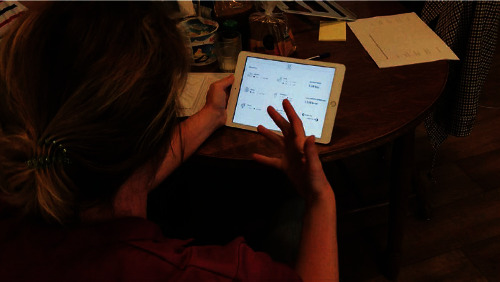
Evaluation of the interactive prototype with the end user.

The interactive prototype was evaluated on a tablet in the nursing home as shown in Figure 5. Individual sessions were conducted with each session lasting for 15 min. The task set out for the participants was to browse through the prototype and gather sufficient data, about a particular PwD, that is relevant to their profession (see Table 2). This is because each profession has a unique role in personalised BPSD management. The participants stopped the task when they thought they had gathered sufficient data. The participants were then asked to think about when they would like to use the digital platform in their working routine, that is, when they would like to analyse the collected data. Field notes were taken and analysed by an affinity diagramming method specific for evaluating interactive prototypes [28]. Building on the feedback, the interactive prototype was improved in terms of data visualisation and interface design and incorporated with real data. A hypothetical case was created to help the care team imagine what the digital platform could do in the long term. User scenarios were created based on when the care team would like to analyse the collected data.

#### 2.3.4. Phase 3

A case study on data analysis was performed using the latest prototype, and the hypothetical case was read to the care team for feedbacks. The care team was asked to select a resident and choose a user scenario, and the data was then collected about this resident for 15 days and provided to the care team. A data collection of 15 days was hypothesised to be able to allow the care team to have an experience of analysing real data within the time limit of the project. The care team was then asked to decide on the most suitable way to analyse these data. In the end, the responsible caregiver of the selected resident and the responsible psychologist analysed the data in a selected user scenario: care plan meeting. The meeting was observed, recorded, and translated from Dutch to English, and notes were taken and categorised using affinity diagramming [27].

## 3. Results

**Figure 6:**
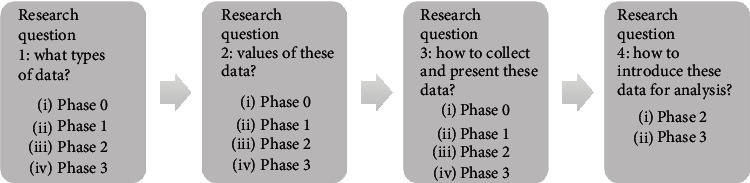
Relationships between the research questions and study phases.

In the following sections, the key findings to the research questions across successive research phases are described. In general, the findings become more detailed and concrete from Phase 0 to Phase 3. In Phases 0 and 1, only the first three research questions were touched upon, because answering the last research question became only possible after the preliminary answers from the first three research questions were available. A reading guide for this section can be found in Figure 6, showing the associations between the four research questions and the phases of the study. It is recommended to refer to Figure 2 for the activities carried out in each phase of the study.

### 3.1. What Types of Data

#### 3.1.1. Phase 0

For the quantitative data, concrete themes and examples of proximal factors were identified from the thematic analysis, which can be found in Table 3. In total, 13 themes were identified and supported by 29 examples. These themes and examples were discussed within the research team to develop a list of parameters that both reflect proximal factors and can be collected by the IPS. The parameters are, for each PwD, over the daytime, “movement distance,” “duration of stay in each room,” and “interaction time with others.” The data directly presenting these parameters are referred to as IPS data in this paper. The IPS data of each PwD are summarised and presented in a few periods, specifically, daily, weekly, and monthly for each member of the care team.

**Figure 7:**
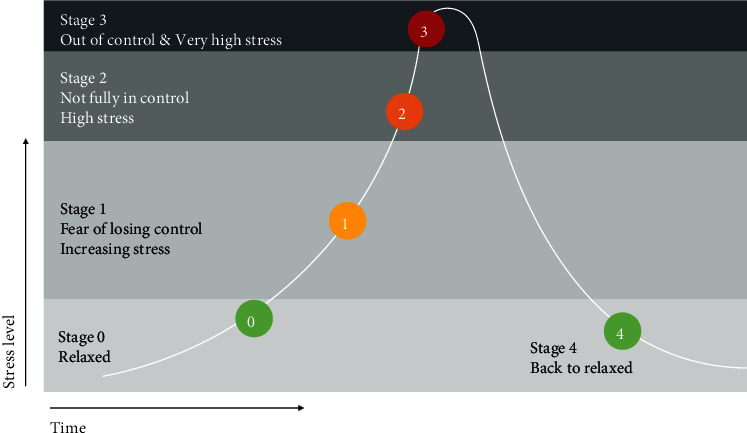
Crisis Development model (Crisisontwikkelingsmodel in Dutch, based on [29]).

For the qualitative data, the nursing home has a reporting system in place for recording behaviours of residents. In this system, the Professional Caregivers write a short report of the behaviours of each PwD each day. In addition, the nursing home has created a crisis-development model (crisisontwikkelingsmodel in Dutch) for each resident (see Figure 7). This model is used to guide Professional Caregivers to take appropriate actions based on the observed behaviours of the PwD [29]. This model is developed based on the observations of a PwD's behaviours for three months, and it consists of five stages, with a colour code for each stage. In stages 0 and 4, a PwD performs usual activities and does not exhibit BPSD (colour green). In stage 1, as PwD's stress level increases, BPSD such as wandering behaviours is observed (colour yellow). In stage 2, the PwD is under high stress and not fully in control (colour orange). In stage 3, the control of PwD is completely gone and crisis events such as physical violence arise (colour red). The short reports and colour codes are of value to reflect the BPSD state of PwD quickly and reliably; thus, they were selected as the qualitative data to be collected. The short reports together with colour labels are referred to as BPSD data in this paper.

#### 3.1.2. Phase 1

During the evaluation of the paper prototype, the Professional Caregivers, doctor, and psychologist pointed out that they would also like to see the movement animations to identify possible movement patterns of the PwD. They gave examples such as “moving back and forth,” “suddenly accelerate,” and “sudden stop,” which are insightful for them to evaluate the behaviours of the PwD.

#### 3.1.3. Phase 2

When evaluating the interactive prototype, the participants found it is time-consuming to watch through the animations for all the residents to identify movement patterns, so they suggested exploring half-hourly IPS data. Half-hourly IPS data is the sum of IPS data for every 30 minutes.

#### 3.1.4. Phase 3

After analysing the real data on the interactive prototype, the Professional Caregiver indicated that she would like to know the sequence of events the resident has experienced over a day, which cannot be captured by the half-hourly IPS data:

*I also want to know the order of events that happened. Now I only know how long she stayed in each room every 30 minutes. But whether she goes to the living room before or after going to the bedroom, I do not know.*

### 3.2. Values of These Data

#### 3.2.1. Phase 0

The care team mentioned that the data collected could be used for detecting the onset of BPSD and effective ways of managing it. As the doctor put it:

*The walking distance. Does it give relaxation? … Because sometimes when people are agitated, they get sort of rest by moving around, but sometimes they get more agitated (because of moving).*

Besides, the care team also thought that the short reports they write could contextualise the IPS data. For example, the travelled distance of the tag could be due to the active movement of the PwD but could also be the result of passive movement, i.e., a Professional Caregiver pushing the wheelchair of a PwD to help the PwD move around. By reading the report, the Professional Caregiver was able to distinguish a passive from an active travelled distance by a PwD and thus provide a well-informed analysis of the physical activity of the PwD.

#### 3.2.2. Phase 1

In Phase 1, during the evaluation of the paper prototype, the participants expressed the more nuanced values of these data to their work, which were different for participants with different professions. The doctor and psychologist reported that they keep “detecting the onset of BPSD and effective ways of managing it” as the main value of data to them. In addition to this value, Professional Caregivers pointed out that they would also use IPS data as a reference when writing the short report:

*I think that we can do better and maybe there's something we can do with the data. We see someone was high in stress. He's walking a lot. We wrote down the distance he had walked instead of “he walks a lot” because 200 meters is a lot to me but 500 meters is a lot to my colleague.*

The manager would like to use data to evaluate the conditions of the residents and decide when they could leave the dedicated BPSD ward and go back to the normal ward, i.e., the ward without frequent BPSD patterns:

*because we are a special ward, only people with behavioural symptoms come here, the caregivers have more time for them (than caregivers from the normal wards). We try to understand their behaviours, and they will be sent back to the normal ward when we find a way to manage these behaviours. So, if we see the “interaction time with others” of one resident has been low over some time, then maybe he could get used to life in the normal ward. We can then send him back to the normal ward and accommodate someone who needs more help.*

Whereas the dietitian stated:

*only the walking distance per day is useful for me, so I can calculate the energy burned by the person.*

Moreover, for the same data type, participants with different professions value the data over different periods; for example, the manager is only concerned about the monthly overview for each PwD, while Professional Caregivers are more concerned with daily data; the manager only wants to get an overview of the BPSD state of a PwD using colour codes, whereas Professional Caregivers need detailed information from the short reports to know what happened to each PwD each day. The doctor and psychologist would like to decide whether or not to read the short reports after reviewing the colour codes.

#### 3.2.3. Phase 2

In Phase 2, when evaluating the interactive prototype, the participants saw the value of data as a reminder. They would like to be reminded to pay attention to a resident when there is a deviation or a sudden change of data collected about this resident. In the busy working environment of a nursing home, with residents who cannot fully express their needs, the care team thinks it is useful if the data can draw their attention to important issues that could be overlooked.

#### 3.2.4. Phase 3

In Phase 3, after analysing the real data on the interactive prototype, the participants identified that the data had the value of understanding the day structure of the residents, which they had not thought about before:

*“we intend to understand more about the behaviours of the resident, but the data is not insightful on that for now, instead, we find it is useful for day structure of the resident”. They expanded: “it is not the purpose (of data analysis), but it is also nice to know their day structure”.*

The insights they generated from the data led to modifications in the care plan. The insights and modifications in the care plan are shown in Table 4.

### 3.3. How to Collect and Present These Data

#### 3.3.1. Phase 0

For collecting IPS data, each PwD participant had to wear a tag during the daytime. As each PwD had his or her own daily routine, daytime was defined as from the moment one is dressed in the morning until one is undressed to go to bed in the evening. Each Professional Caregiver participant agreed to also wear a tag during his or her shift. Each tag had a specific number. The movement distance of each PwD per day was defined as the travelled distance of the tag per day. The duration of stay in each room of each PwD per day was defined as the duration of stay of the tag in each room per day. The interaction time with others of each PwD per day was defined as the duration of two or more tags being physically close per day. Physically close was defined as the distance between the tags being less than 0.5 m for more than one minute. The researchers acknowledge that these definitions are by no means completely accurate in reflecting the activities of PwD; rather, they acted as starting points for the researchers and the care team to link IPS data with the activities of PwD which reflect proximal factors for BPSD.

**Figure 8:**
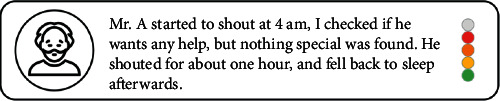
The interface page allowing Professional Caregivers to colour-code short reports by clicking on the drop-down list.

To collect BPSD data from Professional Caregivers and keep track of and present all the data, the concept of a digital platform was developed. The digital platform was designed to be used on a type of tablet which is under current use in the nursing home. In terms of collecting qualitative data, this platform allows Professional Caregivers to write short reports and save all the reports. Currently, there is no obvious link between the short reports and colour codes in the working practice of Professional Caregivers. Therefore, in this platform, Professional Caregivers are required to judge in which phase of the crisis-development model the PwD is, given the behaviours of the PwD recorded in the short reports. This is achieved by letting Professional Caregivers give a colour label for each short report in the platform (Figure 8).

In terms of presenting data, familiar forms of data visualisation for Health Care Professionals and Professional Caregivers were chosen, such as bar charts, pie charts, and line charts. This is because some Professional Caregivers said they have difficulties with interpreting complex data visualisation. The short reports were gathered on an overview page and ordered based on when they were created.

#### 3.3.2. Phase 1

While evaluating the paper prototype, all participants indicated that they would like to see IPS data and BPSD data being presented on the same page; in this way, it would be easier for them to draw inferences. For example, by looking at the combined data of BPSD state and walking distance for a PwD over a few weeks, they would be able to judge if there is a correlation between these two parameters (“correlation” is used in a broader sense in this paper, i.e., the tendency for two parameters to change together in either the same or opposite direction, instead of the definition in statistics). All participants indicated that they would like to make some notes and highlights on the digital platform to record their insights when analysing the data and share the notes with the care team.

At the end of Phase 1, an interactive prototype was developed. Considering the varied values of data expressed by different professionals when evaluating the paper prototype, the interactive prototype was developed to be able to prompt the participants to select their professions and then present the type and period of the data according to their professions. As the study progressed, the types and periods of data presented to each profession on the prototype were updated according to the findings in each phase (see Table 5).

**Figure 9:**
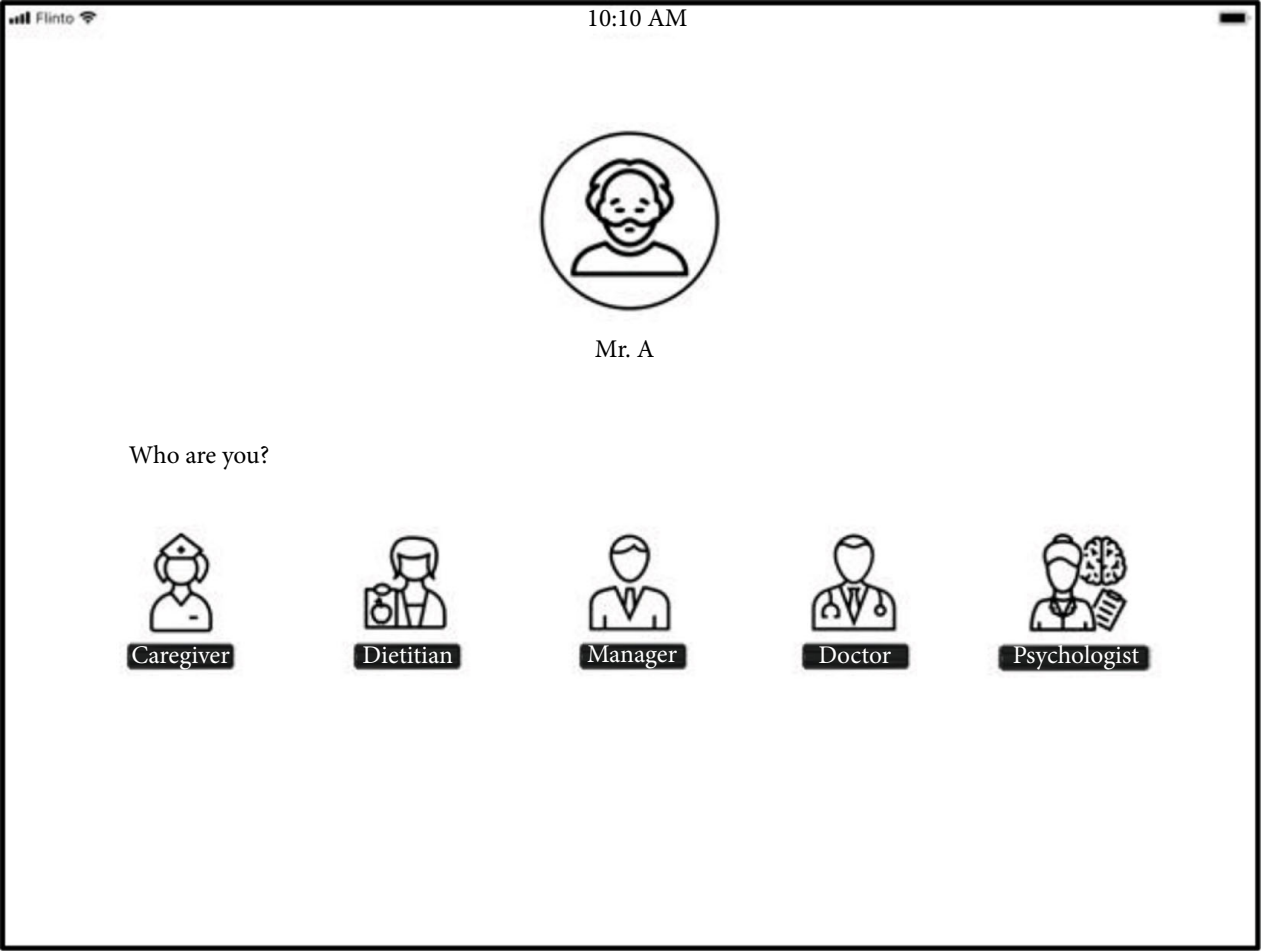
Home page of the digital platform prototype.

**Figure 10:**
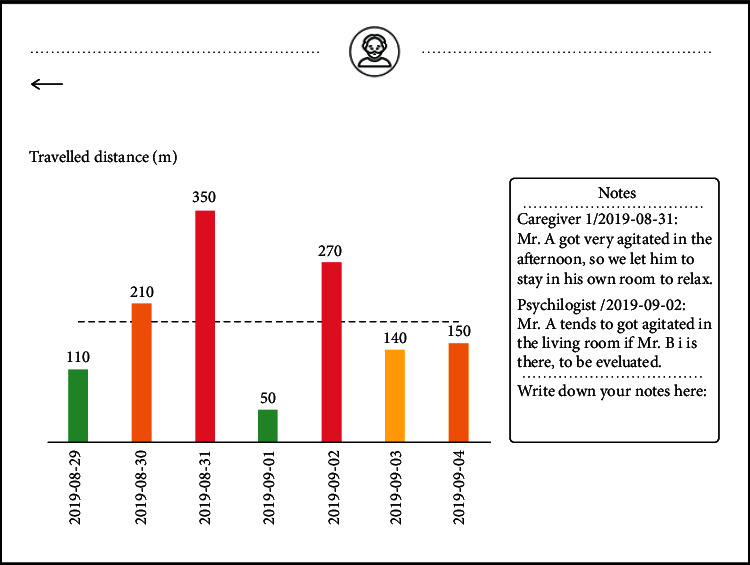
The interface page presenting the distance data and BPSD state of a PwD. The Health Care Professionals and Professional Caregivers can write notes on the right.

The home page of the digital platform is shown in Figure 9, in which the five professions of the care team, i.e., Professional Caregiver, Dietitian, Manager, Doctor, and Psychologist are presented. The movement patterns are added to the prototype and presented in the form of animations. The IPS data and BPSD data are combined in the prototype. For example, the travelled distance data in a week are presented in a bar chart. The height of the bar presents the daily travelled distance, and the colour of the bar presents the colour label given to the PwD each day. This interface page is shown in Figure 10. On each page, Professional Caregivers and Health Care Professionals are encouraged to write short notes and make highlights. All the notes are then collected on an overview page which is accessible to the whole care team. The participants can click on the notes on the overview page to go back to the page on which the note was made. On this page, they can examine the original IPS data and BPSD data accompanying the note. It is worth to clarify that the digital platform has two overview pages for qualitative data, one for short reports, and the other for notes.

#### 3.3.3. Phase 2

After evaluating the interactive prototype, all participants would like the digital platform to prioritise the presentation of the data according to the urgency of the events the data represents; for example, the data showing a PwD is in a bad condition (e.g., suddenly reduced movement distance by half) is more urgent than showing a PwD is in a good state (e.g., colour label stays green for a week). The participants stressed they still would like to know both deterioration and improvement in BPSD for each PwD. All participants found push notifications would be helpful to remind them to use the digital platform, and the notifications should only show urgent events.

Based on the feedback from Phase 2, the interactive prototype was improved in terms of data visualisation and interface design. Since the notification feature requires collecting data for more than one month, which is time-consuming, a hypothetical case was created to help the care team imagine how the future digital platform could help with personalised BPSD management. The hypothetical case is shown in Table 6.

#### 3.3.4. Phase 3

While collecting real data, the main difficulty of collecting data was identified to be the unestablished working routine for Professional Caregivers to use the tags. Out of the 15 days, 3 days of IPS data were completely missing because the Professional Caregivers forgot to give the tag to the resident and 4 days of IPS data were partially missing because the tag was not fully charged the night before. This insight pinpoints that there are several elements involved in data collection, and all elements, including tag usage, should be designed carefully to ensure high-quality data to be collected. Despite incomplete data, the Professional Caregivers have identified the value of the IPS in understanding the day structure of PwD, which informed the modifications of the care plan as mentioned above.

**Figure 11:**
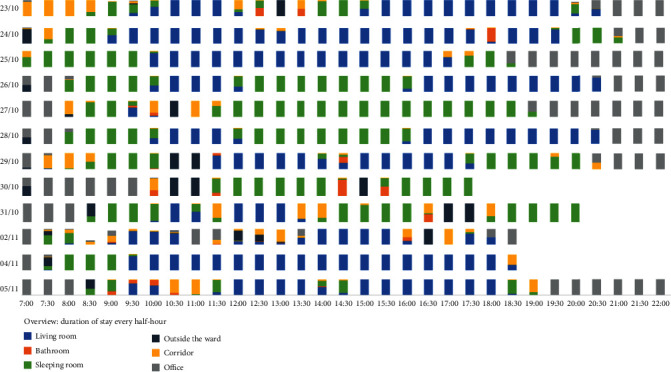
The overview page of IPS data represented to the Professional Caregivers (the tag was charged in the office after being used, hence long duration of stay in the office at the beginning and end of the day; the white space indicates data are partially missing on Oct 30, Oct 31, Nov 2, and Nov 4).

In terms of data visualisation, the half-hourly Indoor Positioning System data over the 12 days (excluding the 3 days with completely missing data: Nov 1, Nov 3, and Nov 6) is summarised on an overview page (an example of “duration of stay” is shown in Figure 11). The Professional Caregivers found the visualisation in the overview page is easy to understand and used it as the guide for reviewing BPSD data:

*First, I look at the overview page, and I find she has lots of time in the living room, then I check what happened in the short report. Normally, if she is in a good mood, she stays in the living room; otherwise, she stays in her (bed) room.*

### 3.4. How to Introduce These Data for Analysis

#### 3.4.1. Phase 2

As mentioned above, we could only begin to answer the last research question from Phase 2 onwards (see also Figure 6). After evaluating the interactive prototype, three user scenarios were identified for introducing data analysis into the working routine of the care team:

1. Before a shift starts: before a Professional Caregiver starting a shift, she/he wants to know if she/he has missed something important in the days when she/he is not in the ward. She/he will then use the digital platform, and since her/his colleagues have highlighted the important things for each resident, she/he can quickly go through all the highlights. If there is a note she/he does not understand well, she/he will click into it to see the data and look up who wrote the note. The notes and data give more details to the Professional Caregiver on what has happened in the ward to keep her/him up to date.
2. When a notification pops up: a notification will pop up if there is a deviation or sudden change of the data collected for a resident. The notification is only seen by the Professional Caregivers. The Professional Caregivers will then open the notification and examine the data. By looking at the data, the Professional Caregivers can determine if anything urgent has happened to the resident based on her/his experience. If she/he thinks actions need to be taken by the care team to address this issue, she/he will organise a meeting with Health Care Professionals via email with the data file attached.
3. Before a care plan meeting: a care plan meeting is about evaluating the care plans of residents by the care team. Before the meeting, the Professional Caregivers and Health Care Professionals will review, independently, the notes on the overview page, and then the data collected. They write down their insights to be discussed in the meeting. The notes and data could trigger the memories of Professional Caregivers and thus help them to better reflect on what has happened during their shifts. Moreover, Health Care Professionals, who were mainly depended on the reports from Professional Caregivers before, could now get access to objective quantitative data to triangulate the information they obtained from the Professional Caregivers.

#### 3.4.2. Phase 3

In Phase 3, during the data analysis, only the Professional Caregiver examined the data before the meeting; the psychologist indicated she was too busy to look at the data beforehand. The Professional Caregiver wrote her insights from data analysis in the form of bullet points. During the meeting, the Professional Caregiver discussed her insights with the psychologist, and the psychologist looked at the data with guidance by the Professional Caregiver. They discussed what to change in the care plan, and the psychologist made her notes (on modifications of care plan) in a notebook. After the meeting, the Professional Caregiver took the notes made by the psychologist and modified the care plan for the resident in an electronic medical record system on the computer. The Professional Caregiver reported that it took her one hour to analyse the data, and she is not confident about her analysis:

*It is a lot of work for me to do. Maximum 30 minutes is ideal for me to prepare the meeting, and my interpretation of the data could be different from my colleagues.*

Therefore, to facilitate the next iteration of data exploration in the future, the aim is to design the data analysis process to be more collaborative and allow users to extract useful information from the data as quickly as possible. Both the Professional Caregiver and psychologist found the hypothetical case was helpful for them to imagine how the digital platform could facilitate BPSD management in the long term.

## 4. Discussion

This paper presents an exploration process of combining quantitative data from the IPS and qualitative data from the care team for facilitating personalised BPSD management in nursing homes. To answer the research questions proposed, the Research through Design approach was adopted and preliminary answers to the questions were generated via developing and evaluating a digital platform. From the literature review, it has been found that the approach of combining quantitative and qualitative data have been used by researchers to understand BPSD, yet the user group of this approach has not been extended to the care team in the nursing home. In comparison to previous studies, the current study led to detailed insights into how the care team would like to combine the quantitative and qualitative data in personalised BPSD management and how they might use these combined data to improve personalisation of care for PwD with BPSD. Specifically, what types of data can be combined, what are their values, how to collect and present them, and how to introduce them in the working routine of the care team were investigated. The methods used and the findings generated in the study are discussed below.

### 4.1. Reflection on Methods

As to the methods, developing a prototype enables different professionals in the nursing home to explore what data and how to collect and present them are of value in real-world conditions for personalised BPSD management. The value of an explorative prototype in the early design stage has been shown by a study in exploring parent-tracked baby data in interactions with Health Care Professionals [30]. Babies cannot fully express their needs and are cared for by parents who need to communicate with Health Care Professionals regarding the past and current state of their babies. Likewise, PwD are cared for by Professional Caregivers who communicate with Health Care Professionals about their BPSD state and interventions. The preferred types and periods of data become more specified for each profession of the care team along with the development of the prototype, as is shown in Table 5.

The researchers also learned that real data could be introduced to the care team as early as possible in the research process. Despite the data collected being incomplete during the technology development, thus not rigorous in the researchers' opinion, they are still insightful for the care team. A previous study has found that discussions about the data become more detailed, personal, and concrete if the data is real and belongs to an individual [31]. When there is no real data available, the researchers discovered that concrete examples of proximal factors could help the research team to decide what parameters derived from the data would be relevant to BPSD management, and interviewing the care team is an effective method to gather these examples.

### 4.2. What Types of Data

Concerning what types of data to be combined, there is a balance among "the insights that could be generated from the data", "the time required to collect and analyse these data", and "the feasibility of the technology". The types of data to be combined can be unique for each PwD. The European Rosetta project identified the usefulness of developing algorithms for monitoring the movement patterns of PwD to detect if there are significant changes in their day-to-day pattern of living at home [32]. This study extends this finding by discovering that deviating from the daily pattern for one PwD who likes wandering (reflected by reduced walking distance) might be different from that for another PwD who is immobile and constantly asking for help (reflected by shorter interaction time with others). Therefore, this study serves as an important step in designing assistive technologies, which is to personalise what types of data can be combined and analysed for each PwD before developing the algorithms. The relations identified by the care team among the different data types are by no means statistically significant correlations or trends; instead, they serve as starting points for explorations. The care team can explore these relations with the methods that they find suitable.

### 4.3. Values of These Data

With regard to the values of data, the data has been found to be valuable for detection, reference, evaluation, reminding, and understanding the day structure of PwD. These values, even when not shared by all members of the care team, are interrelated. This is because these values serve the common goal of the care team—managing BPSD in a personalised way. This study also discovered that different kinds of professionals find different types of data or the same type of data but in different detailedness valuable for their work, which corroborates with previous findings [30]. The researchers also identified the quantitative and qualitative data are of value to each other during data collection and analysis. It has been uncovered that quantitative data could improve the quality of qualitative data during the data collection phase. For example, the Professional Caregivers will write more objective short reports, e.g., stating how long a person has walked, based on the IPS data. During data analysis, the qualitative data can be used to contextualise the quantitative data, which has been reported previously [22]. It has been found that the participants tend to start from the overview of the quantitative data, which guide them to select the relevant qualitative data to analyse. The combination of the data could trigger deeper and more detailed insights that the care team is sometimes even unaware of themselves.

### 4.4. How to Collect and Present These Data

To engage Professional Caregivers and Health Care Professionals in data collection, this process needs to be quick and standardised yet flexible. It has been found that building the data collection process on the current working routines of Professional Caregivers and Health Care Professionals could minimise their workload when collecting data. By colour-coding the reports that they have already established a habit to write, the BPSD data collected are standardised. Yet, Health Care Professionals and Professional Caregivers have the flexibility to write notes of any content that they find relevant for their work. Respecting the working routine of Health Care Professionals and Professional Caregivers has been shown to be essential in previous research projects in nursing homes [33, 34]. In the near future, the tag usage could be built into the current working routine of the Professional Caregivers to collect more reliable IPS data.

Regarding how to present the data, this study uncovered the importance of the overview page: the participants used the overview page as the reference page when analysing the data. With the current data visualisation, the participants preferred using the overview page of the quantitative data to that of the qualitative data. The data visualisation on the overview page is critical for the efficiency of data analysis. A low efficiency could lower the willingness for the care team to analyse the data, thus hindering collaboration. Similarly, a study supporting parents in recording and sharing the health information of their babies to the Health Care Professionals found that a comprehensive yet clear data overview could make these communications more efficient [35]. More explorations are needed in improving the overview pages in the future. For the overview page of quantitative data, explanatory data visualisation techniques could be applied [36]; for the overview page of qualitative data, the researchers could explore natural language processing, word clouds, keyword searches, and more sorting methods, such as sorting against colour coding [37]. Previous researchers found that the types of visualisation strongly influenced the discussion with the users [22]. The researchers of the current paper hence plan to also adopt exploratory data visualisation techniques to enable the care team to explore the data in different perspectives in future studies. The same with previous researchers, the researchers in the current study have processed and visualised the data before presenting it to the participants as the data collected is of large quantity. Data visualisation by the research team is a vital step in the early research stage to make the data more understandable to the Health Care Professionals and Professional Caregivers, even though it is inevitable for the researchers to add their interpretations about the data during this process.

### 4.5. How to Introduce These Data for Analysis

Introducing the data into the working routine of the care team for analysis means the care team can analyse and discuss the data collected effectively in their work. The importance of respecting the working routines of the care team is discussed in the data collection stage; similarly, in the data analysis stage, this process needs to be quick and standardised yet flexible. The researchers learned that data should be presented according to the level of urgency to let Health Care Professionals and Professional Caregivers focus on the most urgent issues in their work and be combined appropriately on one page to reduce memory burden for them. User scenarios created based on when and where the care team would like to analyse the data nudge them to analyse the data at fixed occasions, for example, before a care plan meeting. Yet, the care team should allow the fact that the outcome of the analysis can deviate from the original aim: as shown in the case study, the Professional Caregivers did not gain insights in the behaviours of the PwD but learned about the day structure of this PwD instead, which also contributes to personalised BPSD management. If the outcome of the analysis is aimed exclusively at uncovering behavioural insights, the care team might overlook the values of day structure for personalised BPSD management. Researchers in Data-enabled Design advocate that their research questions develop hand in hand with their understanding of the data and intelligent ecosystem [22]. In this study, the questions that the care team would like to answer with data also developed together with their understanding of the data; therefore, this openness is essential in effective data analysis.

### 4.6. Limitations and Future Work

In terms of limitations, the interviews and evaluation sessions were carried out in a one-to-one format in the current study; a potential drawback of this format is that discussions between participants are lacking and these discussions might stimulate more ideas. However, in an individual setting, the participants can express their own needs and wishes, instead of going along with what is expressed by the more dominant participants, which often happens in a group discussion. In addition, the sample size in this study was small, which makes it difficult to generalise the study results. In another nursing home with different participants, the content and design of the prototype might be developed differently. The strengths of this study are that a wide range of professionals was included, and they participated in several phases of the Research through Design process, from contextual inquiry to prototype evaluation. The use of different professionals' perspectives in the development phase of assistive technologies for PwD has been proven valuable [32, 38, 39].

In the near future, the digital platform will be improved based on the feedback in Phase 3 and will be implemented in the nursing home for a few months. In this way, the researchers can gain more insights into the long-term value of the data. This is because the most recent feedback obtained from the care team in Phase 3 was based on the first-time use of the digital platform by the care team, and factors affecting the data collection and analysis, e.g., patience, time investment, and intuitiveness of the interfaces, might change over time. Secondly, sufficient data will be gathered to form baselines for the behaviours of PwD participants, and algorithms will be developed for pushing notifications to the care team. Then, the researchers can evaluate the prioritisation of the data presentation and push notifications, e.g., does each profession have a preferred prioritisation? Thirdly, with a large amount of data collected, the research team can develop machine learning algorithms to identify movement patterns of PwD in the animations, which could be a new data type to be explored with the care team.

## 5. Conclusions

A better understanding of how to combine qualitative data generated by the care team and quantitative data generated by assistive technologies for personalising BPSD management has been gained in this study. The types of data to be combined, their values, how to collect and present them, and how to introduce them for analysis should be carefully thought through when introducing assistive technologies for personalised BPSD management. A Research through Design approach is effective in engaging the care team in reflecting on these questions. Researchers and designers are encouraged to use the findings of this study as the starting point, investigate other types of data collected by assistive technologies, and explore other dementia care contexts to promote personalised BPSD management in the near future.

## Figures and Tables

**Table 1:** Participants involved in location data collection.

Participant type	Number	Inclusion criteria
PwD	5	Diagnosed with dementia; residing in the BSPD ward
Professional Caregivers	12	Working in the BPSD ward

**Table 2:** The number and professions of participants involved in the Research through Design process.

Profession	Phase 0	Phase 1	Phase 2	Phase 3
Professional Caregiver	3	2	6	1
Doctor	1	1	1	0
Psychologist	1	1	1	1
Dietitian	1	1	1	0
Manager	1	1	1	0

**Table 3:** Themes and examples identified for the proximal factors (the number in the brackets indicates the code number used in thematic analysis).

Three aspects of proximal factors given in the Need-driven Dementia-compromised Behaviour model	Themes for each aspect	Examples of each theme
Personal needs	Negative emotional state	Unfamiliarity (1, 4, 5)
		Stress (20)
		Confusion (7)

Personal needs	Unmet physiological needs	Wrong diet (10, 11)
		Hunger and overeating (12)
		Lack of movement (13)
		Lack of sleep (14, 15)
		Toilet needs (17)

Personal needs	A mismatch between functional ability and performance	Lack of freedom of movement (18, 19)
		Break of routine (21, 22)

Physical environment	Unsuitable light level	Not enough light to see clearly (31)

Physical environment	Unsuitable sound level	Too low (23)
		Too high (24, 25, 26, 27)
		Echo (28, 29, 30)

Physical environment	Unsuitable temperature	Too low or high (36)

Physical environment	Number of people in the surroundings	Too many people walking in the surroundings (32, 33, 34)
		Staying by oneself in a room for too long (35)

Physical environment	Smell	Unpleasant smell (37, 38)

Physical environment	Weather	Lack of sunshine (3, 40)
		Bad weather has a negative influence (39)

Physical environment	Interior decoration	Match personal identity (41)

Social environment	Caregiver demeanour	Lack of interaction with PwD (42)

Social environment	Family visit	The contrast during and after the family visit (6)
		Tiredness after a family visit (8, 51)
		A family member could give more rules to PwD (49, 52)

Social environment	Other staff visits	Normally do not interact with PwD (43)

Social environment	Fellow residents	Realising oneself is different from the others (48)
		Disliking the behaviours of other residents (44, 45)
		Physical closeness and interactions are normally negative (46, 47)

**Table 4:** The insights generated from data analysis and the subsequent modifications in the care plan from the care plan meeting.

Insights (input of the care plan meeting, represented by the quotes from the Professional Caregiver)	Modifications in the care plan (output of the care plan meeting)
“If she is not being invited to the living room, she will call somebody, and if she is then being invited to the living room, then the problem is solved. Maybe she is thinking I have finished morning care and eating, why am I still in my room? So, to prevent her from getting high in stress, we should invite her to the living room around 9:30 am.”	Invite the resident to the living room around 9:30 am
“After activities, most of the time she will be brought to her room. From the data, I can see sometimes she is brought to the living room. She tends to get high in stress when she is brought to the living room.” “When she is high in stress, and gets invited to the living room, sometimes she is fine but sometimes she will have stress if she stays in the living room.” “I will ask the physiotherapist to end the session in her room.”	Bring the resident to her room after activities
“There is one day that she had both music therapy and physiotherapy, it is too many stimuli for her.” “There is one time that she went to the activity room with the volunteers, that was too much for her.” “One hour of activity is enough for the day and not too long.”	Limit the number of activities per day
“If she had lots of activities in the day, like visits from the family, going to the church, music therapy, physio, it is better for her to eat in her own room.” “If there are not so many things going on, she then eats in the living room.”	Choose dinner location based on the daily activities
“Sometimes when she is shouting in the living room and being brought back to her room, after a while, she is invited to the living room again, she does not shout anymore, she just mumbles unhappily.” “I will let her stay in her room after activities. It does not need to take long. After around 30 mins, I will check if she is OK. If she needs more time, I will go check after another 30 mins.”	Invite the resident to the living room as soon as she is calmed down

**Table 5:** The types and periods of data presented to participants on the prototype according to their professions (where D = daily data; W = weekly data; M = monthly data; HY = half-yearly data; HH = half-hourly data).

Profession	Paper prototype with pseudo data	Interactive prototype with pseudo data	Interactive prototype with real data
Professional caregiver	Movement distance [D, W, M] Interaction time with others [D, W, M] Duration of stay in each room [D, W, M] BPSD state (short reports and colour labels) [D, W, M]	Movement distance [D, W] Interaction time with others [D, W] Duration of stay in each room [D, W] BPSD state (short reports and colour labels) [D, W] Movement pattern [D]	Movement distance [HH, D, W] Interaction time with others [HH, D, W] Duration of stay in each room [HH, D, W] BPSD state (short reports and colour labels) [D, W]

Doctor	Movement distance [D, W, M] Interaction time with others [D, W, M] Duration of stay in each room [D, W, M] BPSD state (short reports and colour labels) [D, W, M]	Movement distance [W, M] Duration of stay in each room [W, M] BPSD state (colour labels then short reports) [W, M] Movement pattern [D]	Movement distance [HH, D, W, M] Duration of stay in each room [HH, D, W, M] BPSD state (colour labels then short reports) [W, M]

Psychologist	Movement distance [D, W, M] Interaction time with others [D, W, M] Duration of stay in each room [D, W, M] BPSD state (short reports and colour labels) [D, W, M]	Movement distance [W, M] Interaction time with others [W, M] BPSD state (colour labels then short reports) [W, M] Movement pattern [D]	Movement distance [HH, D, W, M] Interaction time with others [HH, D, W, M] BPSD state (colour labels then short reports) [W, M]

Dietitian	Movement distance [D, W, M] Interaction time with others [D, W, M] Duration of stay in each room [D, W, M] BPSD state (short reports and colour labels) [D, W, M]	Movement distance [W, M, HY]	Movement distance [W, M, HY]

Manager	Movement distance [D, W, M] Interaction time with others [D, W, M] Duration of stay in each room [D, W, M] BPSD state (short reports and colour labels) [D, W, M]	Interaction time with others [M] BPSD state (colour labels only) [M]	Interaction time with others [M] BPSD state (colour labels only) [M]

**Table 6:** The hypothetical case on how the future digital platform could help with personalised BPSD management (the hypothetical data collected are highlighted in italics).

Hypothetical case
Mr. A has been diagnosed with Alzheimer's disease (the main disease which causes dementia), and his data has been collected for one month in the nursing home. Through analysis, the Health Care Professionals identified that Mr. A prefers to stay in the *dining room* in the *morning* but in his *bedroom* in the *afternoon*. His *average walking distance* is 200 meters per day. *The optimal duration of interaction* with him is around 30 minutes. If the duration is longer, Mr. A *tends to be tired*; if the duration is shorter, he tends to *go to bed late*. However, one morning, Caregiver B noticed Mr. A *does not want to go to the dining room* and *gets agitated very often*. Since Caregiver B was also busy with other residents, she/he reported this on the digital platform and carried on working. The next morning, a notification was generated in the digital platform showing Mr. A's *average walking distance* had decreased by half, and the *interaction duration* had dropped to 10 minutes. The digital platform suggested a meeting with Health Care Professionals. Because of working in shifts, Caregiver B was not in the nursing home, so Caregiver C organised a meeting with Health Care Professionals to discuss the current state of Mr. A. Based on the data from the digital platform, the Health Care Professionals decided to take Mr. A for a health check and identified that he had a bruised leg. The Health Care Professionals then updated the care plan for Mr. A, which included treatment for the bruised leg, serving food in his room, and informing family members about Mr. A's situation; thus, they would adjust their visiting time. After the care plan update was made, Health Care Professionals were able to evaluate the effectiveness of the updated plan with the new data from the digital platform. In summary, even though Mr. A could not express what had happened to him, the digital platform sent out real-life notification based on the data collected by IPS and Professional Caregivers, and the Health Care Professionals used the data in the digital platform to identify the change in Mr. A's behaviour and respond quickly without Caregiver B's presence. In this way, the quality of care received by Mr. A was ensured. Without the system, Mr. A's change in behaviour might has been overlooked, which might have caused a delay in his treatment.

## Data Availability

The qualitative and quantitative data used to support the findings of this study are restricted by the ethical committees of Delft University of Technology and Zorggroep Elde in order to protect the privacy of the participants. Data are available from the corresponding author for researchers who meet the criteria for access to confidential data.

## Supplementary Materials

Supplementary materials The supplementary file includes the interview guide supporting the study of the manuscript and ethical approval letter.
