# Supplementary material for: Personalising Management of Behavioural and Psychological Symptoms of Dementia in Nursing Homes: Exploring the Synergy of Quantitative and Qualitative Data
Source: Biomed Res Int. 2020 Jul 9;2020:3920284. doi: 10.1155/2020/3920284 (PMC7368953; doi:10.1155/2020/3920284)
Supplement: Supplementary materials — The supplementary file includes the interview guide supporting the study of the manuscript and ethical approval letter. [file 3920284.f1.pdf]

Interview guide

Research questions:

What are the proximal factors for BPSD encountered by the care team?

What are the according examples of these proximal factors?

Introduction:

Hi, I am xx, today I'd like to ask you a few questions about the complex behaviours (BPSD is referred to as complex behaviours in the nursing home) of the clients you encountered (PwD is referred to as clients in the nursing home), this interview will take around 45-60 minutes.

Before we start just to clarify, the comments you make is for research only. We will not assess your performance based on your answers, we want to know how to help you to work better in managing complex behaviours.

Do you mind if I take an audio recording? I will not share it to others, this is just so that I don't miss anything. Thanks.

Introduce the model:

To start with, we would like to introduce you a model developed for explaining complex behaviours [present the printed model to the participant]. This model is called Need-driven Dementia-compromised Behavioural model. This model divides the factors contributing to complex behaviours into background and proximal factors. The background factors are the root causes of the complex behaviours while the proximal factors are the triggers of the complex behaviours. The background factors are inherent to the person thus difficult to change, while the proximal factors could be modified for preventing or intervening complex behaviours. We would like to know more about the proximal factors i

Interview questions:

May you recall a moment you encountered when one client exhibits complex behaviours? What do you think are the triggers for this?

What are the other factors that you think could trigger complex behaviours?

May you give an example for [each factor indicated by the participant]?

[After 25 mins or if the participant has difficulty with thinking of examples:

In the Need-driven Dementia-compromised Behavioural model, the background factors include neurological status, cognitive status, general health, and psychosocial factors; the proximal factors include personal needs, and physical and social environments.]

Do you agree that personal needs could trigger complex behaviours?

What kind of personal needs could trigger complex behaviours?

May you give some examples?

Do you agree that physical environments could trigger complex behaviours?

What kind of physical environments could trigger complex behaviours?

May you give some examples?

Do you agree that social environments could trigger complex behaviours?

What kind of social environments could trigger complex behaviours?

May you give some examples?

Are there any other factors that you think could trigger complex behaviours which are not included in the model?

May you give some examples?

Thank you – those were all the questions I have for you. If anything else occurs to you after today, please don't hesitate to let me know. Do you have any questions? Thanks again!

Date 03-12-2018
Contact person Ir. J.B.J. Groot Kormelink, secretary HREC
Telephone +31 152783260
E-mail j.b.j.grootkormelink@tudelft.nl

Human Research Ethics Committee
TU Delft
(http://hrec.tudelft.nl/)

Visiting address
Jaffalaan 5 (building 31)
2628 BX Delft

Postal address
P.O. Box 5015 2600 GA Delft
The Netherlands

Ethics Approval Application: Managing Behavioural and Psychological Symptoms of Dementia (BPSD) through Design – a field study with indoor positioning system
Applicant: Wang, Gubing

Dear Gubing Wang,

It is a pleasure to inform you that your application mentioned above has been approved.

The application is approved under the condition that all data will be fully anonymized in publications and during presentations.

Good luck with your research!

Sincerely,

Prof. Dr. Sabine Roeser
Chair Human Research Ethics Committee TU Delft

Prof.dr. Sabine Roeser
TU Delft
Head of the Ethics and Philosophy of Technology Section
Department of Values, Technology, and Innovation
Faculty of Technology, Policy and Management
Jaffalaan 5
2628 BX Delft
The Netherlands
+31 (0) 15 2788779
S.Roeser@tudelft.nl
